# Mosaic *VSGs* and the Scale of *Trypanosoma brucei* Antigenic Variation

**DOI:** 10.1371/journal.ppat.1003502

**Published:** 2013-07-11

**Authors:** James P. J. Hall, Huanhuan Wang, J. David Barry

**Affiliations:** Wellcome Trust Centre for Molecular Parasitology, University of Glasgow, Glasgow, United Kingdom; London School of Hygiene and Tropical Medicine, United Kingdom

## Abstract

A main determinant of prolonged *Trypanosoma brucei* infection and transmission and success of the parasite is the interplay between host acquired immunity and antigenic variation of the parasite variant surface glycoprotein (VSG) coat. About 0.1% of trypanosome divisions produce a switch to a different *VSG* through differential expression of an archive of hundreds of silent *VSG* genes and pseudogenes, but the patterns and extent of the trypanosome diversity phenotype, particularly in chronic infection, are unclear. We applied longitudinal *VSG* cDNA sequencing to estimate variant richness and test whether pseudogenes contribute to antigenic variation. We show that individual growth peaks can contain at least 15 distinct variants, are estimated computationally to comprise many more, and that antigenically distinct ‘mosaic’ *VSG*s arise from segmental gene conversion between donor *VSG* genes or pseudogenes. The potential for trypanosome antigenic variation is probably much greater than *VSG* archive size; mosaic *VSG*s are core to antigenic variation and chronic infection.

## Author Summary

*Trypanosoma brucei*—a deadly parasite of humans and animals—owes its success to its ability to cope with host immunity, and the mechanism it uses to do so is a remarkable example of biological variation. Immune responses that develop against the parasite surface coat are only partially effective against the parasite population; some individual parasites will have already switched to a different variant of the coat antigen, and thus survive to prolong infection. Little is known about how the pattern of antigen variation unfolds, particularly after the early stage of infection. Here, we examined different antigen variants that appeared over the course of infection, to estimate their diversity and to see whether the parasites are able to generate new antigen variants by combination. We found antigen diversity was much greater than expected, and that ‘mosaic’ variants—produced by combining bits of more than one antigen gene—played a central role in the later stages of infection. These results provide important evidence for the robustness of this key survival strategy, provide clues about its evolution, and allow us to identify patterns in common with other antigenically variable pathogens.

## Introduction

A survival strategy common to many bacterial, viral and eukaryotic pathogens, comprising the most rapidly evolving arms race between pathogen and host, is antigenic variation [1]. Over the course of infection, the host mounts specific immune responses against a major pathogen surface antigen, but these are unable to eradicate the entire pathogen population, as some individuals have already switched to express different variants of the antigen. As the survivors proliferate, the process reiterates, resulting in chronic infection that favours transmission. Antigenic variation is powered by diversity in expressed antigens across the pathogen population over the course of infection, and reinfection of partially-immune or already-infected hosts, commonplace in many field situations, is also favoured by expressed antigen diversity [2].

African trypanosomes—parasites of humans and animals in sub-Saharan Africa—have perhaps the most comprehensive system of antigenic variation described [3]. Bloodstream trypanosomes are enshrouded in a dense, highly immunogenic coat of variant surface glycoprotein (VSG) homodimers that conceals invariant surface molecules of the parasite and is the major target of the host immune response [4]. Each VSG monomer consists of a membrane-proximal C-terminal domain (CTD) that is inaccessible to antibodies [5], and an exposed N-terminal domain (NTD) that contains the biologically relevant epitopes [6]. Only one *VSG* is transcribed at a time, but spontaneously, and at high frequency (0.1–1% switch/parasite/generation), the expressed *VSG* is changed, usually through its replacement with a different *VSG* ‘donor’ gene *via* gene conversion [7]. The *Trypanosoma brucei* genome accommodates an archive of thousands of *VSG*s, located mainly in subtelomeric arrays on conventional chromosomes [8] and in the subtelomeres of a pool of approximately 100 minichromosomes [9]. The archive of the *T. brucei* reference strain (TREU927/4) is well annotated, but is likely to remain somewhat incomplete, due to poor coverage of the minichromosomes and the fact that often only one of each pair of homologous chromosomes is represented. Bringing the genomically encoded diversity present in the archive to bear on a host would favour prolonged infection [10]. However, most annotated archive genes are pseudogenic, with only an estimated 5% of the array *VSG*s predicted to be fully functional [11]. Furthermore, infections tend to be dominated by the non-switching, quiescent, ‘stumpy’ trypanosome transmission form, which could further limit expressed antigenic diversity [12].

Many pathogens, including *Anaplasma* spp. [13], *Borrelia burgdorferi* [14], *Neisseria gonorrhoeae* [15], *Treponema pallidum* [16], *Mycoplasma* spp. [17] and *Babesia bovis* [18], undergo a process of segmental gene conversion (SGC) that introduces variation in the expressed antigen. In this process, conversion occurs within the open reading frame, producing a gene that contains segments from two or more donors. By varying only the immunodominant region of an antigen, SGC can make efficient use of a small genome, and can potentially generate vast combinatorial diversity from a limited ‘archive’ of antigen genes [19]. *VSG*s can also undergo SGC. In its simplest form, *VSG* SGC replaces just the NTD-encoding part of the gene, retaining all or part of the previously expressed CTD-encoding region [20], [21]. In other cases, SGC occurs throughout the *VSG*, producing ‘mosaic’ genes [22]–[24], which tend not to appear early in infection and may be selected by immune responses as infections progress [25]. It has been hypothesized that ‘strings’ of related mosaic *VSG*s, produced stochastically by the accumulation of SGC events in a sublineage, could produce novel variants, facilitating both prolonged infection and superinfection of partially-immune hosts [26]. However, as most previous work has focussed on the early stage of infection, the patterns, extent and function of *VSG* expression in the chronic stages are still unclear.

How is *VSG* switching mediated in chronic infection, what is the extent of expressed antigenic diversity, and to what degree does mosaicism contribute to the diversity phenotype? We have sequenced and analysed hundreds of cDNAs harvested longitudinally from 11 chronic infections to identify the prevalence and patterns of mosaicism, and have subjected a ‘string’ of expressed mosaics to serological analyses. Our results show far greater richness in *VSG* expression than previously thought, and demonstrate that mosaicism is a major contributor to chronic infection.

## Results

### Segmental gene conversion frequently contributes to *VSG* variation

To follow changes in *VSG* expression, RNA was purified from blood samples collected longitudinally from 11 mice infected with *T. brucei* TREU927/4 GUTat 10.1. *VSG* sequences were retrieved by *VSG*-specific cDNA amplification, cloning and sequencing, rather than *via* next-generation RNA sequencing, the short read-lengths of which would have complicated unambiguous assembly, especially in a background of expression of related *VSG*. In total, 756 full-length and 8 partial *VSG* sequences were obtained, and each sequence was assigned a three-part name XX-YYcZZ, where XX was the infection number, YY was the sampling time in days, and ZZ was a numerical identifier. These data were supplemented with data obtained from similar infections [11], to give 801 sequences.

**Figure 1:**
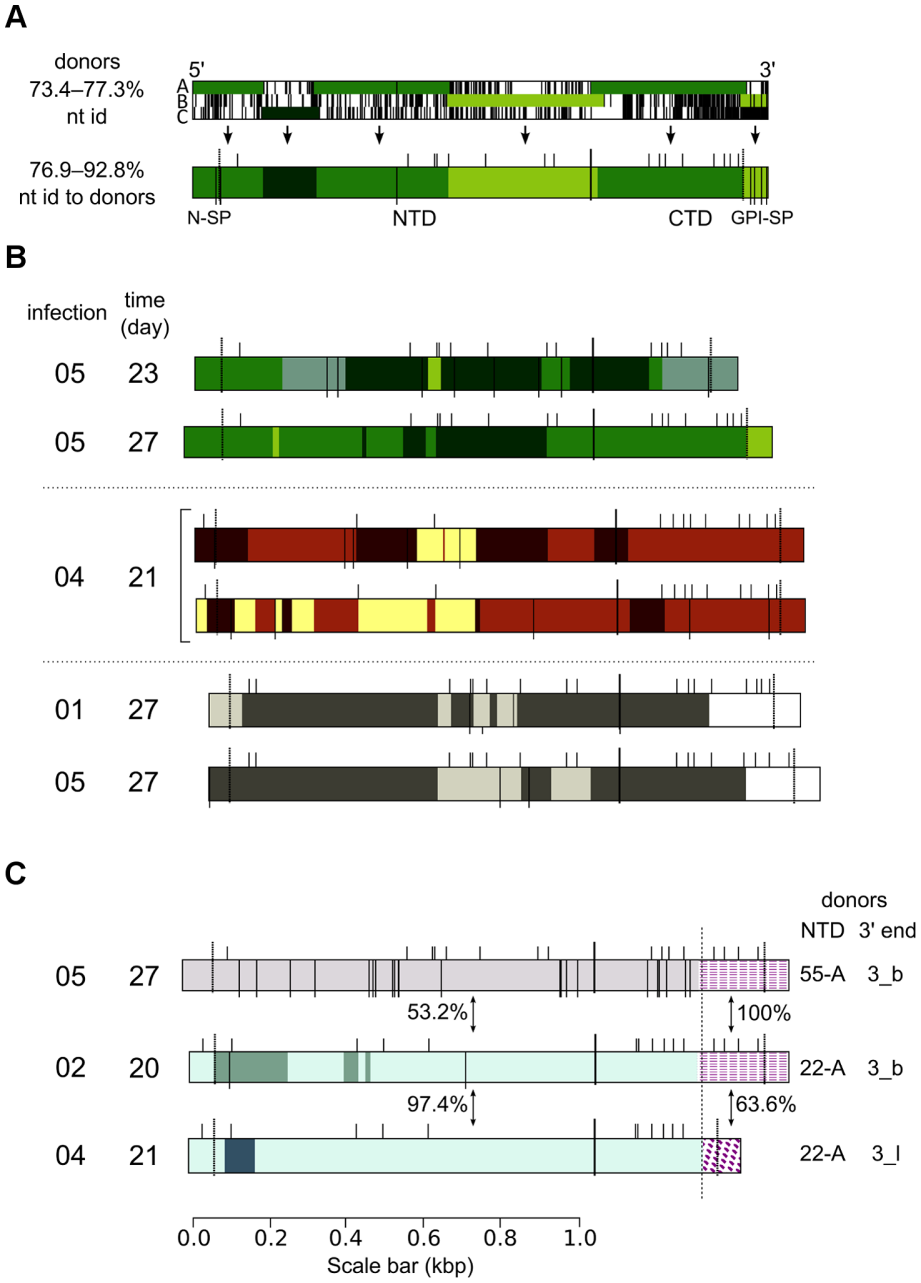
Segmental gene conversion occurs readily during infection. (A) The top diagram represents a multiple sequence alignment between clone 03-32c07 and its three putative donors 14-A (A), 14-B (B) and 14-C (C). The diagram runs 5′ to 3′ left to right. Mismatches between the clone sequence and each individual donor are indicated by black bars. The most parsimonious pattern, minimising the number of contributing segments and mismatches, is highlighted, and is summarized in the lower diagram. Segment contribution was inferred when there was >1 nt difference from the donor contributing surrounding segments. In the lower diagram, dotted and bold lines divide the sequence into the regions encoding the N-terminal signal peptide, the mature NTD, the mature CTD, and the GPI-anchor signal sequence. Black bars projecting from the top of the diagram indicate conserved cysteine codons, and black bars spanning and projecting from the bottom of each diagram indicate the positions of putative point mutations, where the expressed VSG differed from all identified donors. (B) Summaries of six example mosaic *VSG*s, from Set_14 (top), Set_10 (middle), and Set_04 (bottom). Diagrams were drawn as in (A). Different colours represent segments contributed by different donors; no donor sequence data was available for the regions coloured in white (3′ donations in Set_04). (C)Summaries of three 3′ donation events. Diagrams were drawn as in (A). Pairwise nucleotide identities between the expressed VSGs, 5′ and 3′ of the boundary of 3′ donation (indicated by the long dotted line), are given (%).

Putative donor genes were identified by comparing sequences with a database of genomic *VSG* sequences (based on www.vsgdb.net, [27], see Materials and Methods) using BLAST [28]. SGC was inferred when two or more donors appeared to contribute to the expressed *VSG* sequence in a segmental fashion, and no other sequences were a more parsimonious match. An example is given in Figure 1A. Expressed *VSG* sequences were also compared with one another. Based on similarities between NTD-encoding regions, the 801 sequences grouped into 93 distinct ‘sets’, each of which was likely to have been founded on a particular primary donor, or group of donors. SGC within a set was inferred when set members were >2.5% divergent from one another in a nucleotide alignment, differences were grouped in one or more clusters (five or more differences over 30 nt), and distinct clusters of differences were observed in different clones. Donors contributing to a set were given a shorthand name xx-y, where xx was the set number, and y a single letter identifier A–D (Table S1).

Donor sequences combined in various ways, generating an additional layer of diversity amongst expressed *VSG*s, as can be seen in Figures 1B and 1C. SGC occurred in two broad patterns: ‘3′ donation’, in which variation from the primary donor occurred in the predicted CTD-encoding region and utilized donors with low overall identity [20], [21], and ‘mosaicism’, which occurred in the NTD and/or CTD-encoding regions of the *VSG* and utilized highly sequence-related donors [11], [22]–[24]. Mosaicism and 3′ donation were often detected in the same clone sequence. Comparison with donors identified mosaicism in 187/629 (30%) unique sequences, and identified 3′ donation in 358/629 (57%) unique sequences. The extent of 3′ donation varied, and in 90 cases (25% of all 3′ donations) the boundary of conversion occurred merely in the region predicted to encode the GPI-anchor signal sequence. For these analyses, 172 sequences were removed as they were incomplete or duplicates of other sequences from the same sample. Comparison between clone sequences identified patterns of variation corresponding with mosaicism in 24/93 sets (26%), and variation at the 3′ end in 32/93 sets (34%).

**Figure 2:**
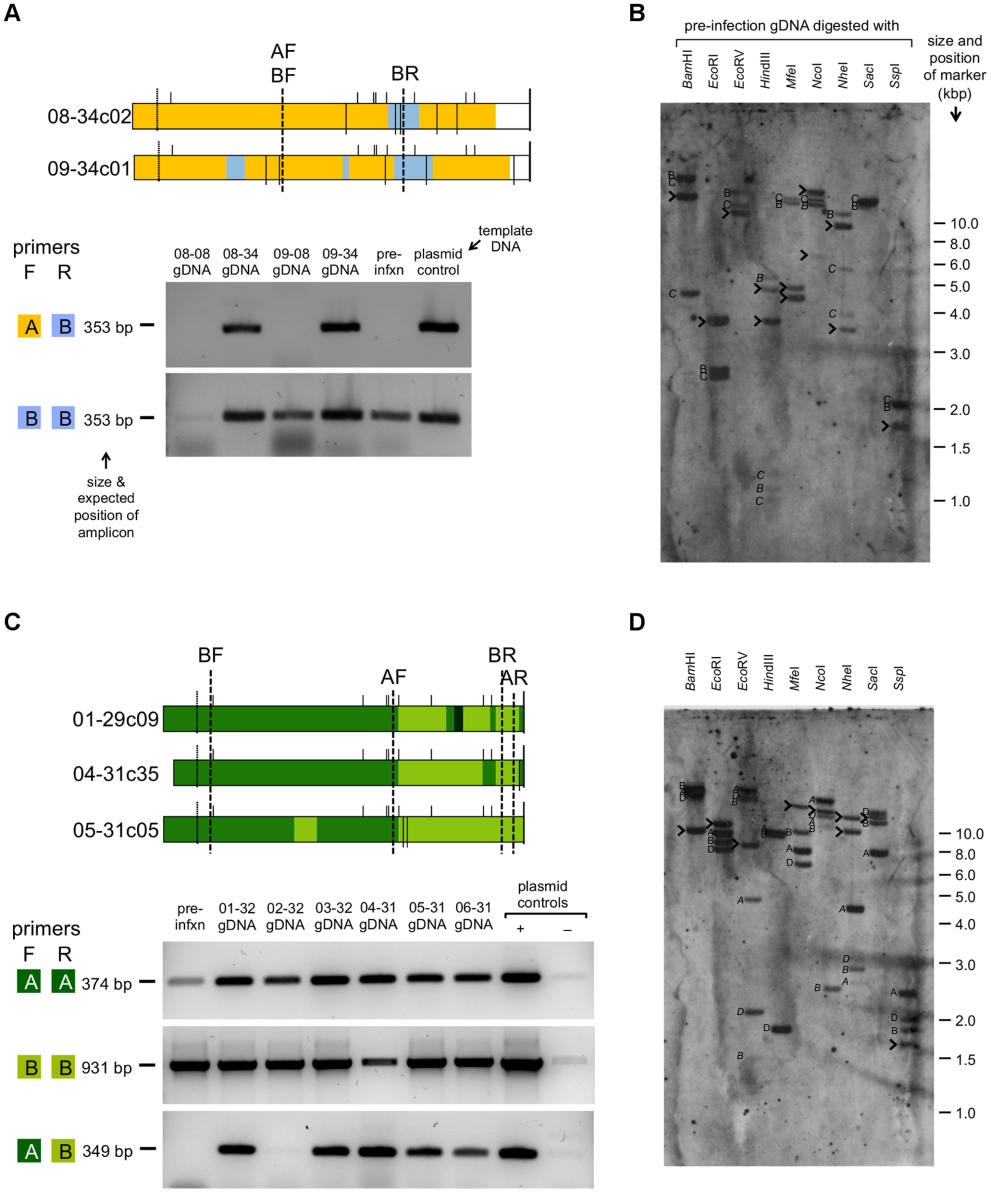
Testing segmental gene conversion events. (A) Top, diagrams of Set_17 clone sequences drawn as in Figure 1, with the binding locations of specific primers for donors 17-A (AF/AR) and 17-B (BF/BR) indicated. Below, PCR was performed using gDNA from pre-infection parasites (pre-infxn), and the first parasitaemic peak (08-08 and 09-08) and terminal samples (08-34 and 09-34) of primary clonal infections, using primer combinations AF-BR or BF-BR; the appearance of the AF-BR product only in the terminal gDNA reactions suggests that the junction A-B appeared over the course of infection and was present in the parasite population gDNA. Isolated plasmid clones were used as positive controls for each reaction. (B) Pre-infection gDNA was endonuclease digested using one of nine enzymes. Gel-separated, blotted DNA was hybridized to a probe corresponding to donor 17-A. Besides donor 17-A, this probe was expected to bind to two other *VSG*s, 17-B and 17-C. The expected positions of fragments corresponding to donors 17-B and 17-C are indicated on the figure with the letters ‘B’ and ‘C’ respectively; where the letter is in italics the fragment binds only part of the probe (hence resulting in multiple bands with a weaker signal). The remaining band(s) are highlighted with arrowheads; the strength of the signal indicates that these likely correspond with donor 17-A, the genomic locus of which is undetermined. (C) Set_14 PCR. Figure is laid out and annotated as panel A, with the binding location of primers specific for donors 14-A (AF/AR)and 14-B (BF/BR) [11] indicated. PCR was performed using gDNA from pre-infection parasites (pre-infxn) and terminal samples of infections (01-32, 02-32, 03-32, 04-31, 05-31, 06-29), using primer combinations AF-AR, BF-BR and AF-BR. Isolated plasmid clones were used as positive and negative controls for each reaction. (D) Set_14 Southern Hybridization using a probe corresponding to donor 14-A. Besides 14-A, this probe was expected to bind to at least three other *VSG*s, 14-B, 14-C and 14-D. Figure is annotated as panel B, with the expected positions of fragments corresponding to donors 14-A, 14-B and 14-D indicated ‘A’, ‘B’ and ‘D’ respectively. In this case it is likely that the remaining band(s) correspond with donor 14-C, the genomic locus of which is undetermined.

Two possible sources of error were that inferred SGC events occurred artifactually by template switching during *in vitro* amplification by RT-PCR, or that inferred SGC events represented the straightforward expression of unannotated *VSG*s in the genome. Two experimental approaches were taken to test these possibilities. First, pairs of primers were designed to bind specifically to one donor or the other, either side of a sequenced SGC event, and PCR reactions using different combinations of these primers were applied to genomic DNA samples, including samples obtained from primary clonal infections. PCR using primers that were both directed against the same donor were able to amplify product from pre-infection, early and terminal genomic DNA (gDNA) samples, but PCR using primers that were directed against different donors—i.e. corresponding with the SGC event—were able to amplify product only from terminal gDNA samples (Figure 2A and 2C). These results are consistent with the SGC event appearing in the gDNA of the parasite population over the course of infection. Second, to test whether inferred SGC could be better explained by unannotated *VSG* present in the genome at the start of infection, restriction endonuclease-digested pre-infection gDNA was analysed by Southern hybridization with a probe corresponding to a donor for the SGC event in question. Identified genomic copies could account for all detected hybridization events (Figure 2B and 2D). Six examples of SGC events were tested by each approach (Figure 2 and data not shown); the results were consistent with neither type of error having arisen.

Together these results show that *VSG* SGC occurs frequently over the course of a 4–5 week infection.

### Segmental gene conversion repairs pseudogenic donors

The properties of the putative donors were then investigated. The NTD-encoding regions were considered separately from CTD-encoding regions, due to the frequent occurrence of 3′ donation (for the latter see below). BLAST searching and pairwise alignments between clone and donor sequences identified 103 donor genes that had contributed to generate the expressed *VSG* NTDs; these are shown in Table S1. The involvement of a further 29 donors, whose sequences were unavailable, could be inferred by comparison between clone sequences. Identified donors were either annotated copies located in the subtelomeric arrays (‘array donors’) or partial sequences or assemblies of read sequences (see Supplemental Experimental Procedures for full details). Eleven read sequences represent putative minichromosomal *VSG*s. It is possible that the 101 sequences (23 sets) for which no donor could be found also represent minichromosomal *VSG*s, which are underrepresented in the assemblies.

Donors for 3′ donation were more difficult to identify unambiguously, perhaps due to (i) the likelihood of their being telomere-proximal [29], and hence underrepresented amongst annotated *VSG*s; (ii) the possibility that successive 3′ donation events accumulating at an expression site produce a sequence with a composite structure that cannot be dissected [29]; and (iii) the general similarity between VSG CTDs [11]. Donors were therefore sought only when at least 80 bp of 3′ donation was apparent. Seventeen 3′ donation donors could be found, with five additional donors inferred by identifying identical 3′ regions in otherwise unrelated clones. Half of all 3′ donation donors (11/22, 50%) corresponded with minichromosomal reads and/or *VSG*s expressed at an early point in infection, and in five cases, indicated in Table S1, there was sufficient downstream sequence to identify ‘TTAGGG-like’ repeats that occur 3′ of telomere-proximal *VSG*s [30]. These findings are consistent with a model in which 3′ donation exchanges the NTD of the expressed VSG, whilst retaining at least part of the previously-expressed CTD sequence.

**Figure 3:**
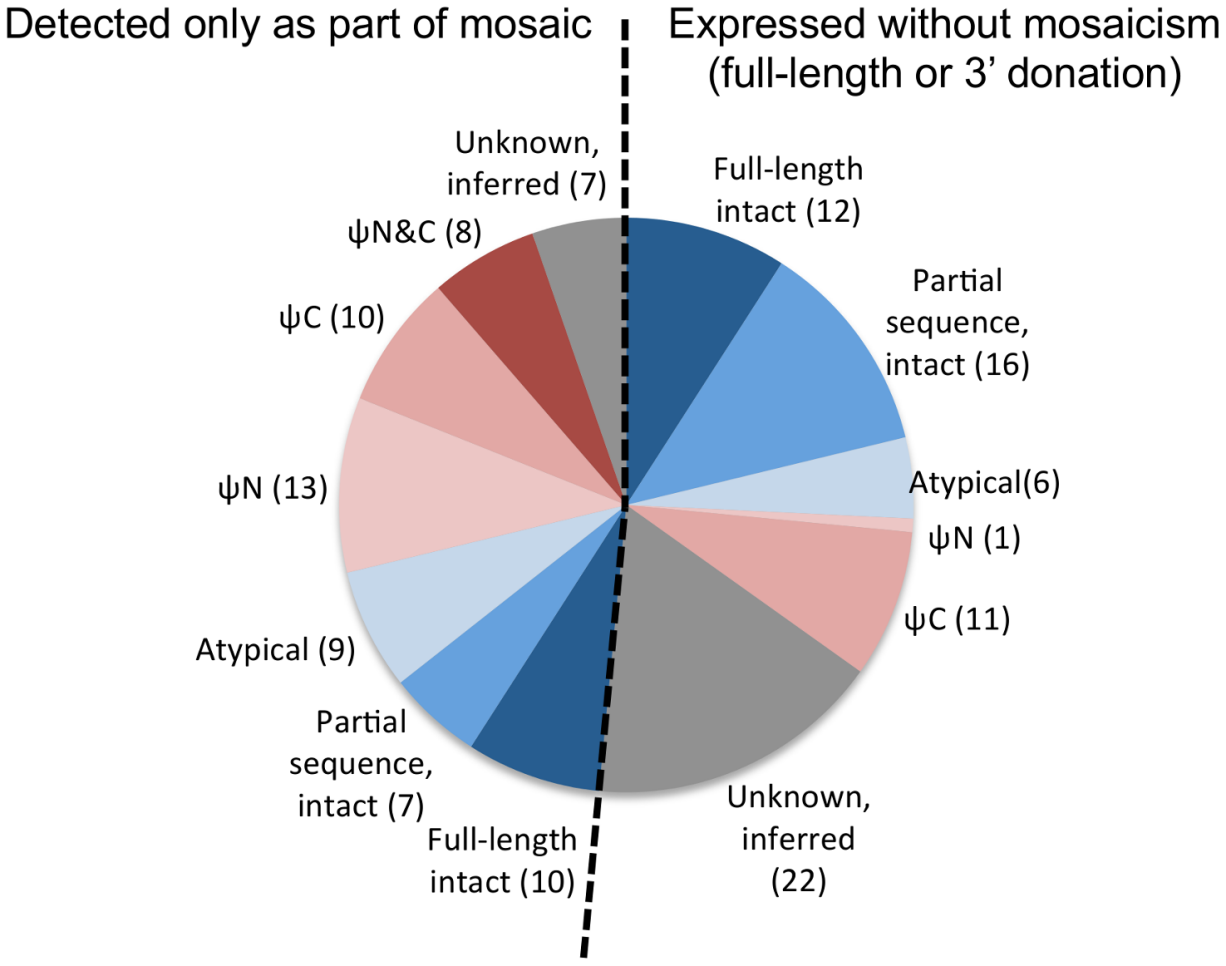
Pseudogene donors were expressed through segmental gene conversion. Of the 103 donors contributing to expressed VSGs for which sequence data were available, 43 were pseudogenes (red). In one case, a pseudogene with damage at the very 3′ end of its NTD-encoding region was repaired by 3′ donation. See also Table S1.

Many (43/103, 42%) of the putative NTD donors were pseudogenes, summarized in Figure 3. Their (partial) expression was achieved by mosaicism, 3′ donation, or both. SGC was thereby able to release genomically-encoded antigenic diversity that otherwise would have been inaccessible.

### *VSG* expression shows great richness and is loosely hierarchical

**Figure 4:**
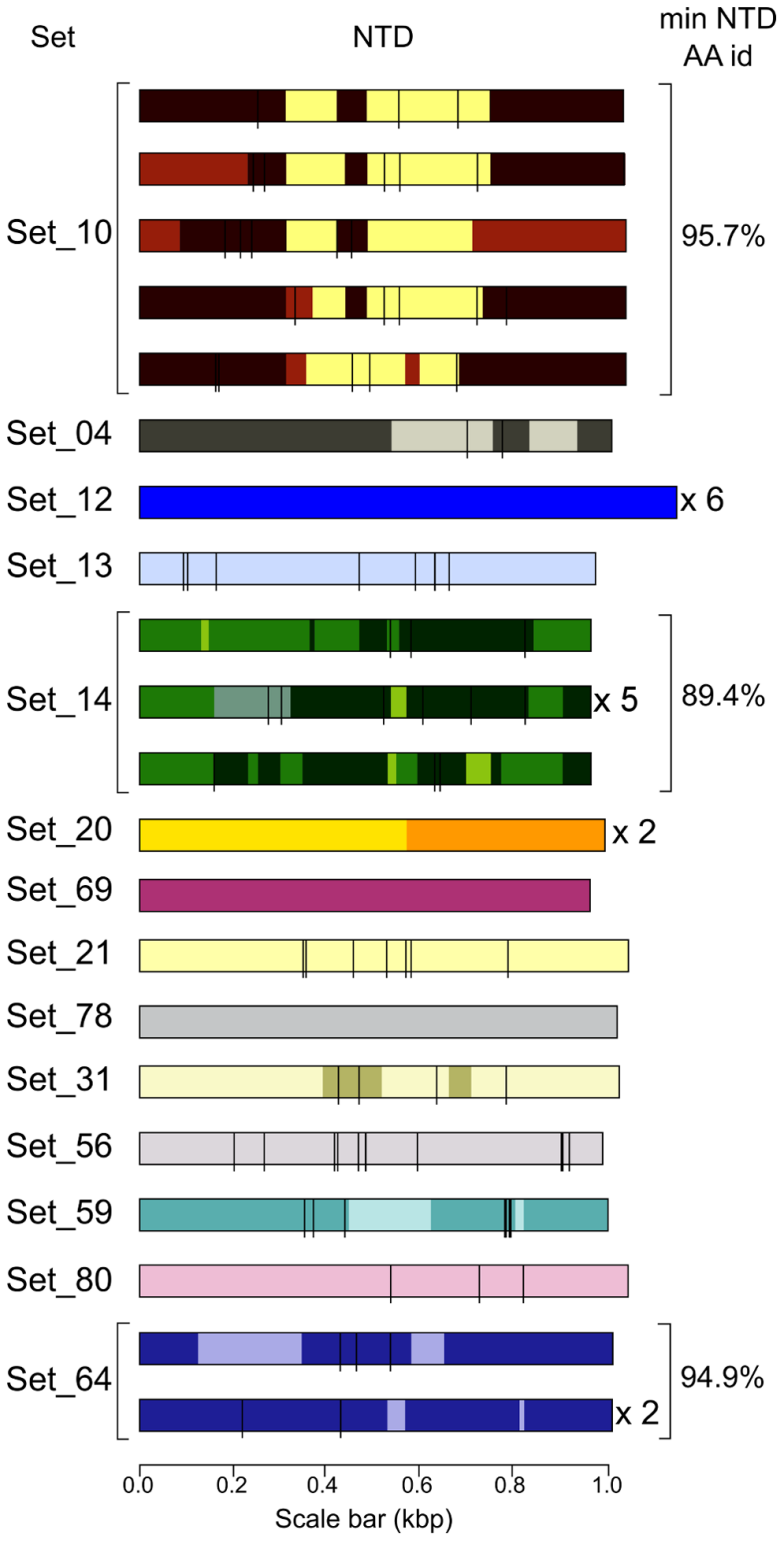
Variants present in an infection. Diagrams representing the mature NTDs of the 32 clones detected at day 27 in the infection of mouse 05. Diagrams have been drawn as in Figure 1A, although here only non-synonymous putative point mutations are included. Where a diagram represents more than one clone, the number of matching clones is shown to the right; variation between clones due to single nucleotide differences is not shown. The pattern of segmental conversion in the Set_20 mosaic could not be determined precisely due to lack of donor sequence data. The clones grouped into 14 sets, three of which showed >1 mosaic variant within this sample. The minimum mature NTD amino acid identity is given for each set of related mosaics. See also Table S2.

‘Richness’—the total number of different variants present in a population—is a principal aspect of diversity [31]. Two infections sequenced to ≥20 clones/sample showed upwards of ten different *VSG* sets in 5/10 samples analysed, as many as 15 *VSG* sets in a sample at one time, and 30–31 different *VSG* sets in total between days 21 and 31. An example from day 27 of infection 05 is given in Figure 4, and further details are given in Table S2. Moreover, the fact that several *VSG* sets were represented by ‘singletons’ (sequence recovered only once) suggests this estimate understates total antigenic richness expressed by the trypanosome population. Rarefaction calculations [32] based on these data project that there could be as many as 95 different *VSG* sets co-present (infection 05, day 27, measured richness 14 sets, Chao1 estimated richness 32.0 sets, 95% confidence upper boundary 95.3 sets). Members of different sets shared less than 59% NTD-encoding nucleic acid identity lending confidence to the premise that richness is immunologically relevant. The relatively small size of many samples makes accurate estimation of total sample antigenic diversity difficult, but it is clear that African trypanosome antigenic variation comprises, rather than homogeneous waves of individual variants, richly diverse populations.

**Figure 5:**
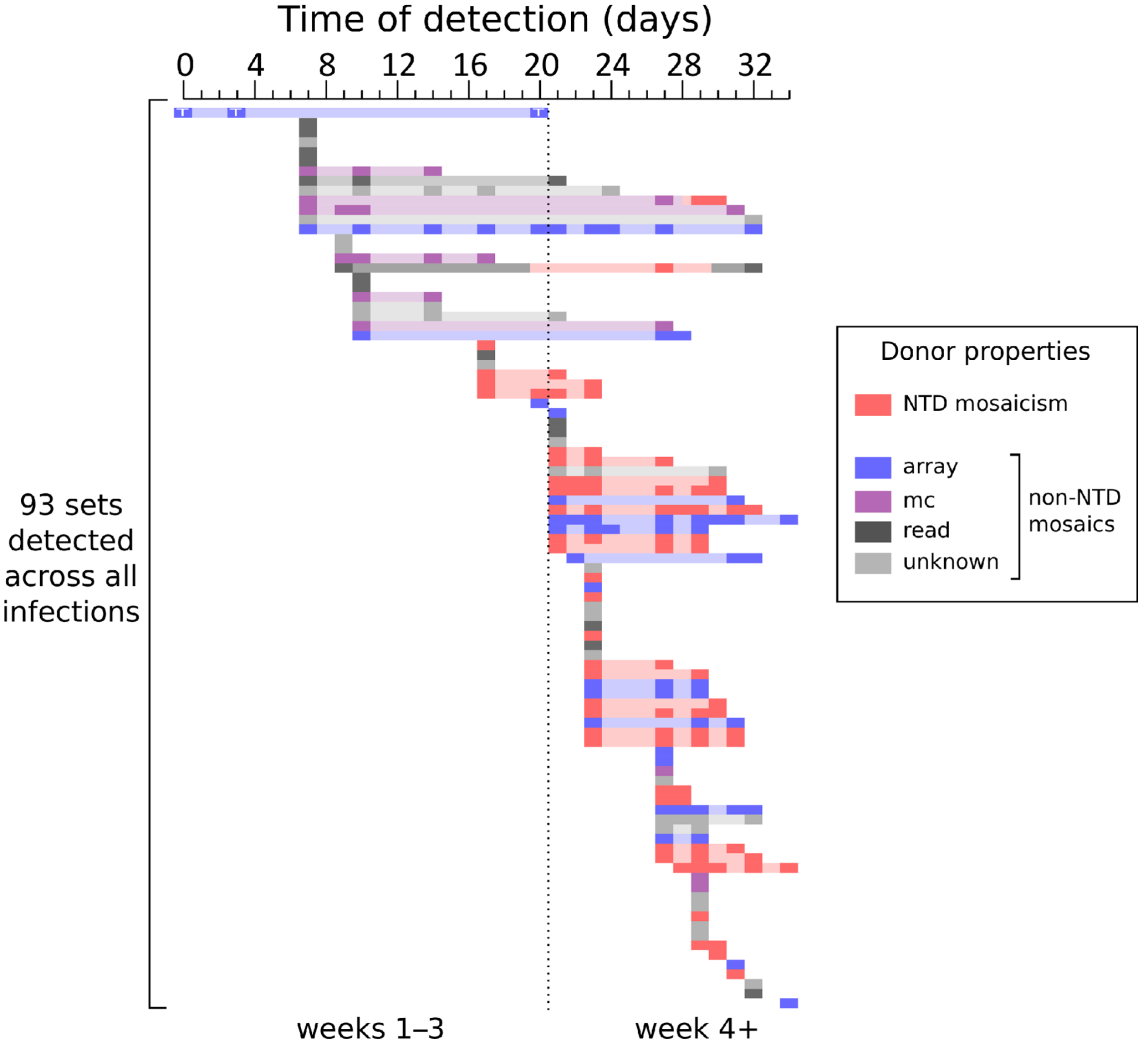
Mosaic VSGs appear later in infection. The times of detection of each of the 93 sets, ordered by time of first appearance, are plotted as dark rectangles, and ranges between earliest and latest times are shown in a lighter shade. Data for each set are coloured according to the properties of its NTD donors. Day 21 is indicated by a dotted line. GUTat 10.1 (Set_01), expressed at the beginning of infection, is known to possess an additional telomeric copy (unpublished data), indicated here with a ‘T’. For two sets, patterns of mosaicism were detected only at specific timepoints and are coloured accordingly. Please note that this figure represents aggregated data from infections initiated with inocula containing varying numbers of T. brucei (see methods).

Despite overall diversity, *VSG* expression followed a loose hierarchy, with the incidence of mosaic and array *VSG*s increasing as infection proceeded (as seen in Figure 5), consistent with evidence from previous studies [7], [11], [33]. Prior to day 21 of infection, only 10/163 (6%) sample-unique sequences (4/24 sets, 17%) were mosaics, compared with 177/466 (38%) of sequences (30/78 sets, 38%) from day 21 onwards. This result held when the more abundant, post-day 20 data were randomly subsampled to 163 sequences without replacement, to control for the differences in number of sequences. Mosaicism is therefore a feature of chronic, rather than acute, infection. However, it is interesting to note that two non-mosaic *VSG*s detected prior to day 21 were closely related to variants that had accumulated segmental conversions in samples obtained from later timepoints (10-07c01 matched mosaics from days 27–30 in infections 01, 06, 10 and 12; 09-03-04 matched mosaics from days 27–32 in infections 01, 05 and 06), indicating that early-expressed *VSG*s can be modified by SGC as an infection progresses.

### Mosaicism accumulates rapidly and introduces an additional level of diversity into expressed *VSG*s

For a given expressed *VSG*, SGC donors shared homology with each other. Mosaic donors had at least 73.2% nt identity (Table S1), but there was apparently no demand for strict sequence identity: in 49 out of 496 mosaic SGCs analysed, the boundary of conversion occurred in a region with less than 4 bp perfect identity between donors, and in three cases (SGCs in 04-21c40, 04-21c04 and 04-27c03) there was 0 bp perfect identity at the boundary. For this analysis, identical SGCs present in different *VSG*s obtained from the same infection were counted only once. Where they could be identified, 3′ donors showed local homology at the boundary of 3′ donation (>85% nucleotide identity over 13–143 bp, median 57 bp), although full-length nt identity was as low as 33%.

Diversity generated by SGC was abundant, even within a single sample. Day 27 in infection 05, for example, saw five related Set_10 mosaic variants, three related Set_14 mosaic variants, and two related Set_64 mosaic variants, shown in Figure 4. A total of seven related Set_22 mosaics were found in infection 04 at day 21. Related mosaics, formed from the same set of donors, had as low as 78.1% amino acid identity (04-23c07 and 04-27c21), although in all cases related mosaics formed from the same set of donors were more similar to one another than their donors were to one another (data not shown).

Progressive mosaicism, in which serial SGC events are proposed to accumulate gradually in an expressed *VSG*, generating an increasingly complex ‘string’ of mosaics, could be inferred in Set_04, Set_14, Set_40 and Set_84. However, predecessors for many complex mosaics, for example 05-27c28, 11-17c01 and 01-27c08, (each constructed from >10 segments) were not identified. Such predecessors may not have been recovered by the process of cDNA cloning and sequencing due their relatively low abundance in a rich population of *VSG* variants, although one would expect a large pool of predecessors to be necessary (and hence readily detected) were each segment being added at maximally the ‘full-length’ *VSG* switching rate of approximately 10^−3^ events/cell/generation [34].

These patterns indicate a role for mosaicism in combining families of related donors—whose members may or may not be intact genes—to generate rapidly an additional layer of combinatorial diversity amongst expressed *VSG*s.

### Related mosaics can be antigenically distinct

Because of the homology between mosaic donors and their products, we selected a string of related mosaic *VSG*s isolated from a single infection (Set_14 from infection 04) to test whether diversity introduced by mosaicism could contribute directly to antigenic variation. These variants had as low as 79.1% amino acid identity between mature NTDs, and each could be explained by the segmental combination of up to four donor genes, 14-A (Tb927.11.20570/Tb11.09.0005), 14-B (Tb927.11.19190/Tb11.13.0003), 14-C (identified in an assembly of read sequences, cloned and sequenced from gDNA and given GenBank accession number KC434956) and 14-D (Tb10.v4.0009), and up to eight independent point mutations. In one case (variant 04-27c44), the expressed *VSG* had also undergone a 3′ donation event. Three of the four donors were pseudogenes; the fourth had an atypical GPI anchor signal sequence with uncertain functionality, as denoted in VSGdb (this signal sequence did not appear in any Set_14 clones).

**Figure 6:**
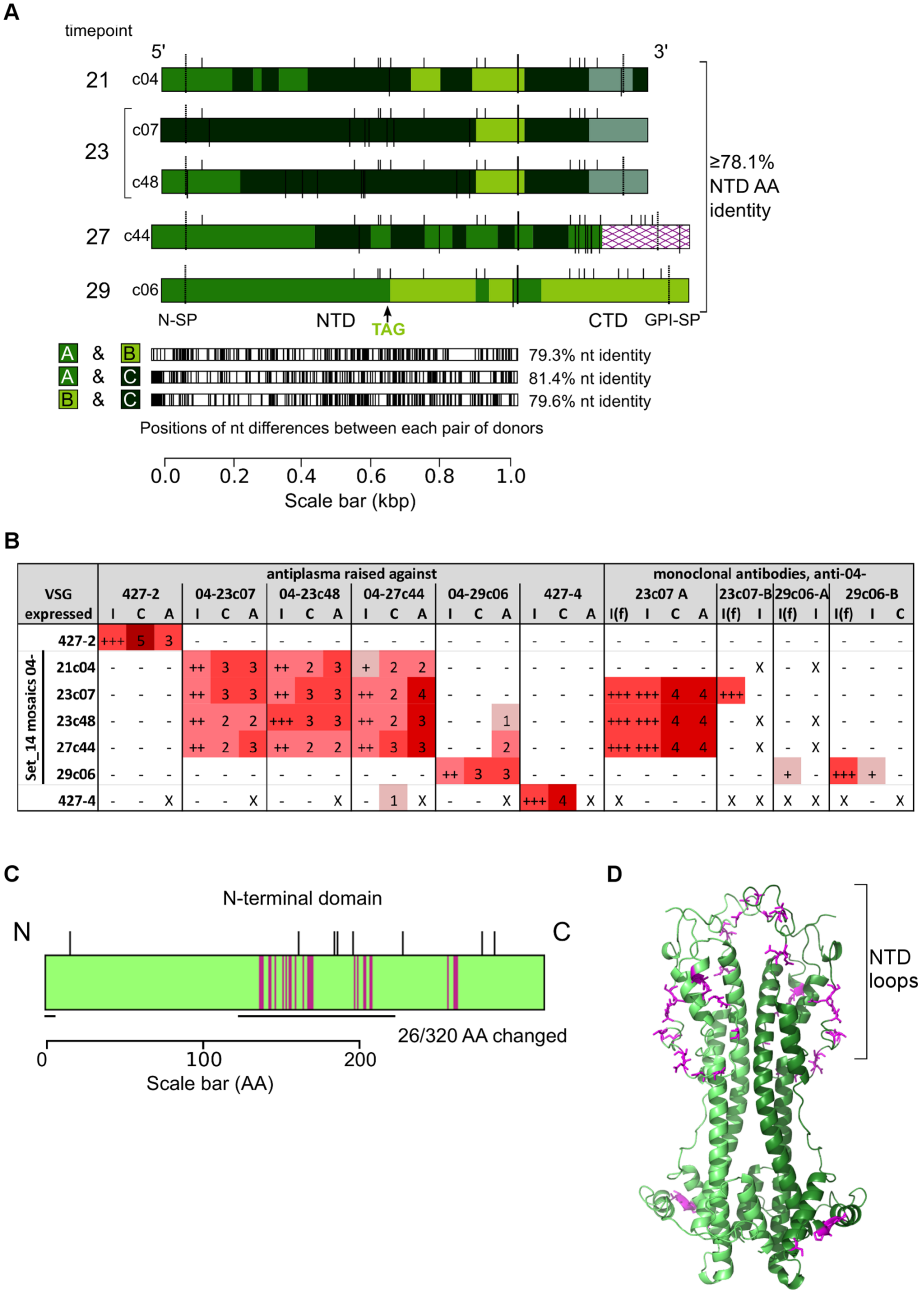
Related mosaics from the same infection are antigenically distinct. (A) Five related Set_14 mosaics from infection 04, variants 04-21c04, 04-23c07, 04-23c48, 04-27c44 and 04-29c06, were drawn as in Figure 1. Below, the locations of differences between each pair of donors. ‘TAG’ indicates the position of an in-frame stop codon in donor 14-B. Comparisons with donor 14-D are not shown due to minimal contribution of 14-D to these mosaics. (B) Results of serological analyses. I = live cell immunofluorescence; C = complement-mediated lysis; A = agglutination; I(f) = acetone-fixed cell immunofluorescence. For the immunofluorescence analyses, ‘+++’ = strong ‘eggshell’-like signal; ‘++’ = strong fluorescence with patchy/posterior accumulation; ‘+’ = weak fluorescence. For CML and agglutination assays, the score is the maximum number of 3-fold antibody dilutions able to give a signal (>95% death/agglutination) when added to an equal volume of trypanosome suspension. ‘−’ = no signal; ‘X’ = test not performed. Each live immunofluorescence combination was performed with at least two antiplasma, CML and agglutination assays were performed with at least one antiplasma, with representative results shown. Some non-reciprocal cross-reaction occurred at higher antiplasma concentrations: suboptimal VSG coverage may have transiently exposed invariant surface antigens, which could be targeted by antibodies. (C) Location of differences in variant 04-29c06 NTD amino acid sequence. The 26 positions where variant 04-29c06 was different from the earlier appearing Set_14 mosaics are indicated by magenta bars. Bars projecting from the top of the diagram indicate conserved cysteine residues, and black bars below the figure indicate the predicted location of NTD loops. (D) The predicted 3D-structure of a variant 04-29c06 dimer was visualised in PyMol (The PyMOL Molecular Graphics System, Version 1.5.0.4 Schrödinger, LLC.). Each monomer is coloured a different shade of green, and the residues represented in panel 5C are coloured purple.

Five of the Set_14 *VSG*s from infection 04, shown in Figure 6A, were expressed transgenically under drug selection as intact surface coats in Lister 427 trypanosomes, as described in Materials and Methods and Figure S1. VSG 427-4, a known functional *VSG* absent from TREU 927/4, was expressed in a similar manner as a negative control. A standard infection-and-cure protocol was used to raise polyclonal antibody responses in mice. At least two different antiplasma were obtained for four of the five variants, as well as for parasites expressing 427-4 and unmodified parasites expressing VSG 427-2 (antiplasma could not be obtained for variant 04-21c04 as this transgenic parasite line exhibited inadequate virulence, data not shown). Monoclonal antibodies (mAbs) were also generated for two of the variants, 04-23c07 and 04-29c06. To test the antigenic relatedness of the Set_14 mosaics, antibodies were applied in three assays on live cells: indirect immunofluorescence, complement-mediated lysis (CML), and agglutination. The results are shown in Figure 6B. With polyclonal antisera, four of the five related mosaics cross-reacted in all assays, reciprocally, but one variant, 04-29c06, which had arisen later than the others in infection 04, was antigenically distinct. Likewise, neither of the anti-04-29c06 mAbs bound to the other four mosaics. One mAb raised against 04-23c07 bound two other Set_14 mosaics, and the other bound only a hidden epitope on 04-23c07, as revealed by acetone fixation; neither bound to 04-29c06.

To investigate the regions of variant 04-29c06 that contribute to its antigenic distinctness, the amino acid sequences of the cross-reacting variants were compared with the sequence of variant 04-29c06. At twenty-six positions in the NTD, shown in Figure 6C, variant 04-29c06 differed from all of the earlier-occurring variants. Predictions of the three-dimensional structure of variant 04-29c06 using I-TASSER [35] and PHYRE2 [36], shown in Figure 6D, suggested that 22 of these residues occurred in the region likely to form loops at the membrane-distal end of the NTD, a region which, on another VSG, correlated with B-cell epitopes [6].

SGC can therefore contribute directly to antigenic variation during infection, by generating related, but antigenically distinct, mosaics.

## Discussion

Antigenic variation is a survival strategy driven by the expression of antigenic diversity by the pathogen population. With their huge archive, rapid switch rate, and large population size within a host it is perhaps not surprising that *T. brucei* infections display great antigenic richness: here we show that many variants, numbering at least 15 in some cases, and estimated to comprise many more, may be expressed across the parasite population at one time. Hosts larger than mice—in which trypanosome antigenic variation likely evolved—are capable of sustaining a greater parasite burden, precipitating even more switch events [37], and thus even greater richness. MacGregor *et al.* (2011) have predicted that, for trypanosomes, the high prevalence of the non-switching stumpy form during chronic infection might limit the expression of different *VSG*s. Our observation of great richness suggests that any such limitation is unlikely to be of significant impact. Conversely, stumpy form prevalence might actually enhance persistence of minor variant subpopulations, by suppressing their numbers below the threshold required for induction of a specific immune response [10], [38]. Trypanosome antigenic variation should be viewed more as stochastic, continuous onslaught by many variants, rather than fastidious and tightly regulated expression of few variants, although it is interesting to note that of the ∼1000 *VSG*s that constitute the annotated archive [11], <10% were identified as contributing to the expressed *VSG*s studied here. By underpinning chronicity of infection, expressed *VSG* diversity likely goes hand-in-hand with the dynamics of differentiation, enhancing opportunities for successful transmission and facilitating the persistence of the trypanosome in its ecosystem [39]. Richness in expressed surface antigen variants may be a feature common to many pathogens, pre-empting host immune responses and memory: antigen sequences cloned from infections by the bacterium *Borrelia burgdorferi* showed non-saturating richness [14] (although some sequences varied only in single nucleotides), and in *Plasmodium* it is likely that the whole archive of ∼60 genes has appeared by day 11 of infection [40].

How does SGC serve the *T. brucei* diversity phenotype? Following the initial phase of infection, associated primarily with non-SGC activation of minichromosomal *VSG*s and distinct peaks of parasitaemia [33], segmentally-converted *VSG*s become abundant. Two broad patterns of *VSG* SGC were observed. One, termed 3′ donation, involves retention of at least part of the previously expressed CTD. Swapping just the antigenically important NTD allows the expression of *VSG*s with damaged CTD-encoding regions—in this way it is analogous to the patterns of variable cassette exchange seen in the variable surface antigens of other pathogens [41]—but it seems unlikely that any combinatorial diversity introduced by 3′ donation can itself contribute to antigenic variation because the boundaries of conversion occur within the buried CTD [5]. The other pattern, mosaicism, can likewise utilise damaged *VSG*s, and also introduces diversity into the antigenically important NTD, generating sets of related mosaics within an infection, and generating infection-unique variants that could potentially contribute to superinfection [26]. Evidence for progressive mosaicism—the stepwise increase in complexity of an expressed mosaic ‘string’ within an infection, similar to that in *Anaplasma marginale* [42]—was limited: the sheer number of different variants present may have prevented detection of intermediate mosaics, but it is also possible that mosaicism is a rapid process, with multiple segments accumulating in a short period. A useful, novel mosaic is a VSG able both to form a functional coat and escape circulating immune responses: rapid SGC, allied with efficient selection—the death of individual trypanosomes that have activated a dysfunctional mosaic *VSG* or perhaps even a form of VSG quality control [43]—would enable a sublineage to efficiently explore the space of potential mosaics, favouring production of a variant that fulfils these criteria. In more natural hosts, where the greater number of switches arising from greater population size accelerates the kinetics of antigenic variation, the infection is likely to progress to this phase sooner, as the easily-activated *VSG*s are neutralised and unique variants remain to drive prolonged infection [25].

*T. brucei* homologous recombination depends on substrate length and homology [44], a pattern reflected in mosaic *VSG* construction: donors shared high identity (>73.7% identity), and thus their associated mosaics were similar to one another. If only similar sequences combine, how efficient can this process be at introducing antigenic dissimilarity? Multiple segments, and the accommodation of non-identity at their flanks, both of which were observed in mosaic *VSG*s, may compensate for overall similarity. *N. gonorrhoeae* and *B. burgdorferi*, both of which rely on SGC for generating and expressing antigenic diversity, show similar patterns: short conversion events with little or no identity at their flanks [14], [15]. Previous analyses of mosaic *VSG*s found that although their products could escape individual mAbs, they were insufficiently distinct to evade polyclonal antibody responses [24], and a study of related VSGs found antigenic divergence between two variants sharing 70% amino acid identity but cross-reaction between a mosaic and its donor, with which it shared 88% amino acid identity, mostly in the NTD [45]. Here, we found that mosaicism could contribute directly to antigenic variation: polyclonal antibodies raised against the earlier-detected VSGs could bind other earlier-detected variants, whereas the variant detected at the later timepoint, sharing between 79.1–87.5% NTD amino acid identity with the earlier variants, was completely antigenically distinct. The capacity of *T. brucei* antigenic variation may therefore be greater than predicted from the genome sequence. Yet given that four donors were required for the assembly of this mosaic *VSG* set, the yield of merely two distinct variants would appear not to be an efficient use of the archive, nor an effective way to introduce antigenic novelty in and of itself. It is possible that testing of further related mosaics would reveal additional antigenically variant forms, and natural infections, with a more extensive chronic phase, may see longer strings of more distinct mosaics. Severe immunosuppression occurring in the mouse model [46] may have also biased against identifying antigenically distinct variants. On the other hand, incomplete variation through SGC might be sufficient in the context of a complex natural infection, where antibody clearance might operate [47], differentiation and incomplete cross-reaction suppress variants below the levels required to induce potent, specific responses [38], and host immunity is suppressed [48].

Perhaps, for trypanosomes, the true value of *VSG* SGC comes from its ability to accommodate and exploit longer-scale changes arising during evolution of the *VSG* archive. Like many other multi-gene families [49], the archive probably evolves through a process of birth-and-death evolution, in which genes ‘born’ by duplication diversify by accumulating mutations and short gene conversions [50]: while some genes persist intact, others acquire disruptive mutations and ‘die’. Their subtelomeric location promotes rapid mutation of archive *VSG*s [51] while the only occasional expression of each *VSG* results in weak selective pressure per allele [52]. As they diverge, it is likely that intact archive *VSG*s become pseudogenic, and damaged archive genes continue to diversify (L. Plenderleith, pers. comm.). Archive diversification is favourable as it facilitates reinfection, likely to be important in isolated foci where most hosts have already been infected. In these circumstances, second order selection—in which the mechanisms responsible for the evolution and maintenance of a gene family are under stronger selection than the individual family members [53]—would favour an expression mechanism that can cope with the pseudogeneity that would inevitably arise following a protocol of archive hypermutation. The ability to express diverging—and possibly damaged—*VSG*s using SGC expands the effective archive size, increasing the total number of antigenically different variants that the parasite population can muster.

Might other species of African trypanosome, such as *T. vivax* and *T. congolense*, similarly rely on SGC for antigenic variation? *T. vivax* and *T. congolense* have a lower degree of archive pseudogenicity than *T. brucei* [54]. Perhaps tighter bottlenecks in the life cycle of *T. brucei* [55], compared with *T. vivax* and *T. congolense* [56], favour deleterious genetic drift, promoting rapid fixation of pseudogenizing mutations. If the generation of mosaic *VSG*s was a specific adaptation in response to this challenge, the occurrence of mosaic *VSG*s in *T. vivax* or *T. congolense* infections might be much less frequent. On the other hand, mosaic *VSG*s might be prevalent in *T. vivax* and *T. congolense* infections if SGC has evolved primarily as a generator of combinatorial antigenic diversity.

SGC is clearly important in underpinning the diversity phenotype in *T. brucei* chronic infection. Further experiments would identify the contribution of SGC to the kinetics of antigenic variation in natural hosts and the role that mosaicism plays in superinfection, particularly in a field setting, and to establish how combinatorial variations generated by SGC can yield antigenically unique variants. In addition, data from *T. vivax* and *T. congolense* infections are required to unravel the selective pressures that have favoured the development of such a comprehensive diversity phenotype.

## Materials and Methods

### Ethics statement

All studies involving animals were conducted in compliance with the UK Animals (Scientific Procedures) Act 1986 (ASPA) and under the auspices of Licence 60/3760 which was approved by the University of Glasgow ASPA Ethical Review Committee.

### Chronic trypanosome infection

*Trypanosoma brucei* TREU 927/4 GUTat 10.1 parasites were used to infect 6–8 week old female Balb/c (infections 01–09) or MF1 (infections 10–12) mice. Two infections, 08 and 09, were primary clonal infections, initiated with single parasites, the others were initiated with 40,000 (infections 01–06) or 10,000 (infections 10–12) parasites. Blood samples were taken into CBSS containing 5% w/v sodium citrate and RNA produced using the RNeasy kit (QIAGEN).

### Amplification and cloning of *VSG*s

Reverse transcription was performed using the SuperScript III First-Strand synthesis kit (Invitrogen) using oligo[dT] as the primer. cDNA was column purified (QIAGEN) before PCR amplification using Herculase II Fusion (Stratagene) using primers directed against the spliced leader and conserved 16-mer (sequences in Table S3). Amplicons were purified and subcloning was carried out using the TOPO-TA subcloning kit as described previously [11]. For each reaction, a control was performed to ensure that the RNA sample was not contaminated with genomic DNA. The *VSG* coding sequence was assembled from overlapping sequence reads produced from primers corresponding to sequences in the vector. In cases where these reads were insufficiently long to obtain a good quality full-length sequence, reactions were performed to cover the central region of the gene. Genomic DNA (gDNA) was prepared by phenol:chloroform extraction and ethanol precipitation, according to a standard molecular biology protocol. PCRs to test for mosaic *VSG*s were performed using *Taq* polymerase according to a standard amplification protocol; primer sequences are listed in Table S3.

### Sequence analysis and manipulation

Sequences were assembled, visualised, compared and analysed using CLC Genomics Workbench (CLC Bio, Aarhus, Denmark), eBioX (available at www.ebioinformatics.org/ebiox) and custom Ruby scripts. Scripts are available on request. Intact sequences presented in this study are contained in GenBank entries KC434459–KC434954, and full details are available from the Dryad digital data repository (doi:10.5061/dryad.7pc00). Richness estimator was ‘bias-corrected Chao1’ performed using EstimateS (Version 7.5, R. K. Colwell, http://purl.oclc.org/estimates). The ‘genomic *VSG* database’ was obtained by collecting all available *VSG* sequences from TriTrypDB (www.tritrypdb.org using the text search query ‘vsg’ and applying a filter to TREU 927/4 genes) and from VSGdb (www.vsgdb.net, all entries). The list was made non-redundant by removing duplicate sequences. The assembly of read sequences is described in Table S4.

### Generation and verification of transgenic *VSG* expressors

Transgenic *VSG*s were expressed under drug selection in Lister 427 trypanosomes, which have an extremely low rate of switching [57], by first inserting the exogenous *VSG* into the active expression site [58], and then removing the endogenous VSG 427-2. *VSG* expression plasmids, a kind gift from G. Rudenko, were manipulated using enzymes provided by NEB according to the manufacturer's instructions. *VSG*s amplified from the subcloning plasmid were cloned into a variant of p221_PUR117VSG_UTR [58], in which VSG117 had been replaced with an *Sbf*I site. The insert was sequenced to ensure fidelity in cloning. *T. brucei* Lister 427 13-90 parasites were maintained in HMI-9 medium supplemented with 20% FBS and passaged regularly to avoid overgrowth. Transfections were carried out using an AMAXA protocol (T-cell nucleofection buffer, programme X-001). After the first round of transfection media were supplemented with 2.5 µg.ml^−1^ puromycin for drug selection, following the second round of transfection with plasmid pBS_VSG221KO (G. Rudenko, manuscript in preparation) media was further supplemented with 1 µg.ml^−1^ blasticidin. To test *VSG* expression, PCR reactions were performed on cDNA using primers directed against VSG 427-2, the Set_14 VSGs, VSG 427-4, and two other Lister 427 *VSG*s, VSG 427-6 and VSG 427-9, according to a standard *Taq* polymerase amplification protocol (primers are listed in Table S3). In each case, parasites were found to be expressing only the exogenous *VSG* under consideration, as shown in Figure S1A. To test for the presence of other *VSG* mRNA, total *VSG* cDNA was amplified and digested with a restriction endonuclease for which a recognition site was present in the Set_14 *VSG*s and not at a similar position in other expression-site-occupying *VSG*s. The digest yielded products of the expected sizes, leaving little or no residual uncut product (data not shown). Amplified *VSG*s, subcloned and sequenced, were found to match the specific variant under consideration. To test whether *VSG* mRNA was being translated, crude cell lysate from 2.5×10^6^ cell equivalents was analysed by SDS-PAGE (NuPage system, Invitrogen). The size of the variant band corresponded with the predicted size of the exogenous VSG, as can be seen in Figure S1B. For two variants (Set_14 variants 04-23c07 and 04-29c06), the variant band was excised from a gel and subjected to mass spectrometry. In both cases, peptides corresponding with the Set_14 variants were identified by at least one significant match and were the only VSGs identified (data not shown). Together, these analyses indicated that the transgenic parasite lines were expressing the *VSG*s under consideration. Furthermore, the survival of the parasites in complement-competent plasma, as can be seen in Figure 6B, indicated that the transgenic surface coat was functional. To avoid prolonged *in vitro* passaging, stabilates of clones were prepared for subsequent experiments; thawed stabilates were maintained in culture for a maximum of two weeks.

### Generation and testing of antibodies

To generate antibodies against a transgenic surface coat, 1×10^6^ parasites were injected intraperitoneally into a Balb/c mouse, which was treated with 20 mg.kg^−1^ cymelarsen (Rhône-Merieux) when the parasitaemia exceeded 10^7.2^ parasites.ml^−1^. Five to eight days after cure, plasma was retrieved from terminal blood samples by collecting the supernatant from a 10 min centrifugation at 14,000 *g*. Monoclonal antibody-producing hybridomas were obtained by preparing splenocytes from these infections according to a standard polyethylene glycol (PEG) fusion protocol. Hybridoma lines were cloned by limiting dilution at least twice to ensure a pure population of mAb. For indirect immunofluorescence, all reactions were carried out on ice. 1×10^6^ cells were incubated in primary antibody solution (1∶25 dilution of antiplasma in trypanosome dilution buffer [47] or undiluted hybridoma culture supernatant) for 10 min. Cells were fixed in the presence of primary antibody to minimise clearance [47] by the addition of 1 vol 8% w/v paraformaldehyde in PBS and incubating for 10 mins. For each reaction a negative control was included to test for non-specific antibody fixation. Cells were washed twice with PBS, resuspended in secondary antibody solution (Alexa-488 labelled goat anti-mouse IgG, provided by Invitrogen, 1∶500 dilution in PBS+1% w/v BSA), incubated for 15 minutes, washed twice in PBS, mounted on a glass slide and examined using a Zeiss Axioscop 2 microscope. For each reaction, minimally 200 trypanosomes were examined. Indirect immunofluorescence on acetone-fixed trypanosomes was performed as described previously [34]. For complement-mediated lysis, complement-competent plasma, obtained from guinea pig blood by centrifugation, was used to dilute antibodies and trypanosomes. Trypanosomes were at a final concentration of 0.5×10^7^ parasites.ml^−1^, and the reaction was incubated at room temperature for 1 hr before scoring for cell death. For the agglutination assay, parasites were at a final concentration of 1×10^7^ parasites.ml^−1^, antibodies and trypanosomes were diluted in TDB, and scoring took place after 30 min at room temperature.

## Supporting Information

Figure S1 **Transgenic trypanosomes were expressing mosaic VSGs.** (A) PCR was performed on cDNA from cultured parasites using primers specific for either VSG 427-2, the Set_14 mosaics or VSG 427-4. Each reaction was numbered according to the template DNA as follows: 1, positive control (gDNA or plasmid); 2, unmodified 427-2 expressers; 3, 04-21c04; 4, 04-23c07; 5, 04-23c48; 6, 04-27c44; 7, 04-29c06; 8, 427-4. (B) Crude cell lysate was separated using SDS-PAGE. Lanes were labelled as in Panel A. Arrowheads mark the position of the variant band in each lane, the migration of which corresponds approximately to the predicted size of the transgenic *VSG*. The identity of the variant bands in lanes corresponding to 4 and 7 were determined by mass spectrometry.
(TIF)

Table S1 **Details of genomic copies.** All identified donors that contributed to expressed VSGs. The read donors were assembled as described in Supporting Material, and assemblies can be found on the Dryad digital data repository (doi:10.5061/dryad.7pc00).
(XLSX)

Table S2 **Number of clones/sets identified in each sample.** For each sample, the number of clones retrieved (top number) and number of sets they form (bottom number) is given.
(XLSX)

Table S3 **Oligonucleotide primer sequences (5′–3′) used in this study.**
(DOCX)

Table S4 **Details of donors assembled from reads.** Read sequences were obtained from ftp://ftp.sanger.ac.uk/pub/databases/T.brucei_sequences/.
(DOCX)
