# Supplementary figures and images for: Mosaic VSGs and the Scale of Trypanosoma brucei Antigenic Variation
Source: PLoS Pathog. 2013 Jul 11;9(7):e1003502. doi: 10.1371/journal.ppat.1003502 (PMC3708902; doi:10.1371/journal.ppat.1003502)

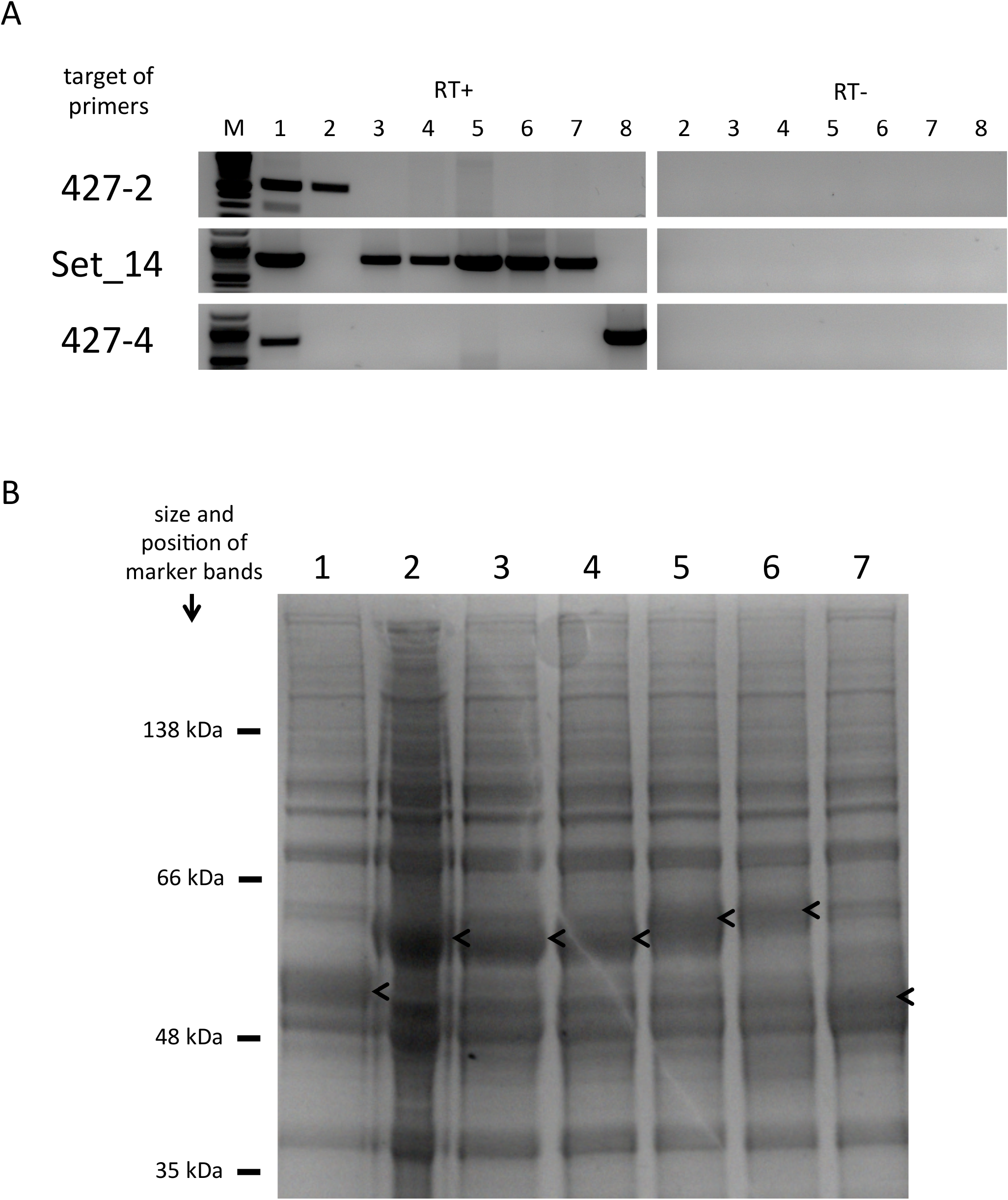

Supplement: Figure S1 — Transgenic trypanosomes were expressing mosaic VSGs. (A) PCR was performed on cDNA from cultured parasites using primers specific for either VSG 427-2, the Set_14 mosaics or VSG 427-4. Each reaction was numbered according to the template DNA as follows: 1, positive control (gDNA or plasmid); 2, unmodified 427-2 expressers; 3, 04-21c04; 4, 04-23c07; 5, 04-23c48; 6, 04-27c44; 7, 04-29c06; 8, 427-4. (B) Crude cell lysate was separated using SDS-PAGE. Lanes were labelled as in Panel A. Arrowheads mark the position of the variant band in each lane, the migration of which corresponds approximately to the predicted size of the transgenic VSG. The identity of the variant bands in lanes corresponding to 4 and 7 were determined by mass spectrometry. (TIF) [file ppat.1003502.s001.tif]
