# Supplementary material for: Mosaic VSGs and the Scale of Trypanosoma brucei Antigenic Variation
Source: PLoS Pathog. 2013 Jul 11;9(7):e1003502. doi: 10.1371/journal.ppat.1003502 (PMC3708902; doi:10.1371/journal.ppat.1003502)
Supplement: Table S3 — Oligonucleotide primer sequences (5′–3′) used in this study. (DOCX) [file ppat.1003502.s004.docx]

| **Experiment** | **Primer name** | **Sequence** |
| --- | --- | --- |
| Amplification of *VSG*s from cDNA | Spliced Leader | GTTTCTGTACTATATTG |
|  | 16-mer | GTGTTAAAATATATCA |
| Testing Set_17 mosaicism by PCR | 17-A_410F | TCGAACGGACAATTGCCC |
|  | 17-B_410F | TCGACCGAAAACTTGCAA |
|  | 17-A_762R | TGTATTTATGTTGTCTTGAT |
|  | 17-B_762R | TGTATTTATGCTGGGTTGGC |
| Testing Set_14 mosaicism by PCR | 14-A_671F | ACGGCCAAACCGGGATGAAA |
|  | 14-A_1024R | ATCGCTTCGCGGACTTCCTC |
|  | 14-B89F | CCATTGTCGACGCAACCGTT |
|  | 14-B657R | GGCCGTCGACACAAAGACAG |
| Oligos to generate *Sbf*I site in p221_PUR117VSG_UTR | FseI/SbfI_5' | ATCAAGCTTGGCCGGCCAGTCCTGCAGGAATTCGAT |
|  | FseI/SbfI_3' | ATCGAATTCCTGCAGGACTGGCCGGCCAAGCTTGAT |
| Testing exogenous VSG expression | Tubulin_F | CCAAGCTCGGCTACACGGTGT |
|  | Tubulin_R | GGCTCAAACACAGCGTTCGA |
|  | 427-2_F | ATGCCTTCCAATCAGGAGGC |
|  | 427-2_R | TGTATCGGCGACAACTGCAG |
|  | Set_14_F | AAAGGAAGGCACAGAAGG |
|  | Set_14_R | GCAGTAATGTCTGATTCG |
|  | 427-4_F | TAAAAGGAGACGGAGTGG |
|  | 427-4_R | GCTCTAGTTTGTTGTTGTTG |
|  | 427-6_F | ACCTGACATCGGACGGTAAC |
|  | 427-6_R | GTCGGTTATGTCGGCAAGTT |
|  | 427-9_F | GTAAACGGTCCGGAGTTCAA |
|  | 427-9_R | CATTTCCGCGTTGTCTTGTA |
