# Supplementary material for: Mosaic VSGs and the Scale of Trypanosoma brucei Antigenic Variation
Source: PLoS Pathog. 2013 Jul 11;9(7):e1003502. doi: 10.1371/journal.ppat.1003502 (PMC3708902; doi:10.1371/journal.ppat.1003502)
Supplement: Table S4 — Details of donors assembled from reads. Read sequences were obtained from ftp://ftp.sanger.ac.uk/pub/databases/T.brucei_sequences/. (DOCX) [file ppat.1003502.s005.docx]

**Table S4.**

| Assembly name | Set | Notes | Reads |
| --- | --- | --- | --- |
| 927mc_III-1b07_assembly | 3 | 3' incomplete; A752del in the NTD-encoding domain of the assembly was corrected according to expressed clones | 927mc_III-1b07.p1k |
|  |  |  | 927mc_III-1b07.q1k-rev |
|  |  |  | 927mc_III-01b01.q1k-rev |
| tryp_X-54b12.q1c-rev_assembly | 4 | 3' incomplete; cloned and sequenced from gDNA (KC434956) | tryp_X-54b12.q1c-rev |
|  |  |  | tryp_XI-910b10.q1k |
| 927mc_VI-6b02_assembly | 5 | 5' and 3' incomplete | 927mc_VI-6b02.p1k |
|  |  |  | 927mc_VI-6b02.q1k-rev |
|  |  |  | 927MC_V-01a09.p1k |
|  |  |  | 927MC_VI-01c10.q1k-rev |
|  |  |  | 927MC_V-01a09.q1k-rev |
| tryp_XI-1034g11_assembly | 14 | cloned and sequenced from gDNA (KC434958) | tryp_IXa-24h09.q1c-rev |
|  |  |  | tryp_XI-1034g11.p1k |
|  |  |  | tryp_XI-1034g11.q1k-rev |
| tryp_XI-934d12_assembly | 16 |  | tryp_XI-934d12.q1k-rev |
|  |  |  | tryp_X-334d06.p1c |
| tryp_XI-1058d01_assembly | 17 | 3' incomplete; cloned and sequenced from gDNA (KC434957) | tryp_XI-1058d01.q1k |
|  |  |  | tryp_XI-1121e11.p1k |
|  |  |  | 927mc_VI-8a07.p1k-rev |
| trypA24a5.p1p | 24 | 5' and 3' incomplete | trypA24a5.p1p |
| tryp_IXa-29b02.q1c_assembly | 25 | 5' incomplete, not contiguous | tryp_IXa-29b02.q1c |
|  |  |  | tryp_IXb-301g08.p1c |
| 927mc_IV-5e11_assembly | 29 | 5' and 3' incomplete | 927mc_IV-5e11.p1k |
|  |  |  | 927mc_IV-5e11.q1k |
| tryp_IXa-10f02_assembly | 32 | 3' incomplete; cloned and sequenced from gDNA (KC434955) | 927mc_IX-10d02.q1k |
|  |  |  | tryp_IXa-10f02.p1c |
| 927mc_VIII-11d07.p1k | 38 |  | 927mc_VIII-11d07.p1k 927MC_VIII.0.118 |
| tryp_XI-1068b03.p1k-rev | 38 | 5' and 3' incomplete | tryp_XI-1068b03.p1k-rev |
| tryp_XI-909g03.q1k-rev | 49 | 5' and 3' incomplete | tryp_XI-909g03.q1k-rev |
| tryp_IXa-20a05_assembly | 51 | 5' and 3' incomplete; cloned and sequenced from gDNA (KC434959) | tryp_IXa-20a05.p1c |
|  |  |  | tryp_IXa-6d11.q1c |
|  |  |  | tryp_IXb-153c03.p1c-rev |
| tryp_IXa-29e12_assembly | 54 | 3' incomplete | tryp_IXa-29e12.p1c |
|  |  |  | tryp_IXa-4a12.p1c |
|  |  |  | tryp_IXb-361b07.p1c |
| tryp_X-99f10_assembly | 54 |  | 927mc_IV-3d11.p1k-rev |
|  |  |  | 927mc_VI-10d07.p1k |
|  |  |  | tryp_X-190c10.q1c-rev |
|  |  |  | tryp_X-214a05.q1c-rev |
|  |  |  | tryp_X-268e08.q1c |
|  |  |  | tryp_X-277b10.q1c |
|  |  |  | tryp_X-338f01.q1c-rev |
|  |  |  | tryp_X-36c03.q1c |
|  |  |  | tryp_X-99f10.p1c |
|  |  |  | tryp_XI-143d03.q1ca-rev |
| tryp_XI-1157a04_assembly | 59 | not contiguous | tryp_XI-1157a04.p1k |
|  |  |  | tryp_XI-1157a04.q1k-rev |
| 927mc_VIII-3d10_assembly | 61 | 3' incomplete, not contiguous | 927mc_VIII-3d10.p1k-rev |
|  |  |  | 927mc_VIII-3d10.q1k-rev |
|  |  |  | 927mc_VIII-13b05.q1k |
|  |  |  | 927mc_VIII-14d10.q1k-rev |
| tryp_XI-1007d10.q1k | 63 | 3' incomplete | tryp_XI-1007d10.q1k |
| tryp_XI-1084f09.p1k | 67 | 5' and 3' incomplete | tryp_XI-1084f09.p1k |
| tryp_IXa-10d01_assembly | 71 | 5' and 3' incomplete | tryp_IXa-25g04.q1c-rev |
|  |  |  | tryp_IXa-10d01.q1c-rev |
| tryp_XI-1009a05_assembly | 72 | 5' and 3' incomplete | tryp_IXa-7a11.p1c-rev |
|  |  |  | tryp_XI-1009a05.p1k-rev |
|  |  |  | tryp_XI-1055f01.p1k-rev |
| tryp_XI-393f08_assembly | 74 | 3' incomplete | tryp_XI-393f08.q1k |
|  |  |  | tryp_XI-949b02.q1k-rev |
|  |  |  | tryp_XI-1098g01.q1k-rev |
|  |  |  | trypA31g9.q1t-rev |
|  |  |  | trypA29a10.p1p-rev |
|  |  |  | tryp_XI-204f04.p1c-rev |
|  |  |  | tryp_X-48f04.p1c-rev |
|  |  |  | tryp_XI-393f08.p1k-rev |
| tryp_IXb-375c10_assembly | 76 |  | tryp_IXb-375c10.p1c |
|  |  |  | tryp_IXb-62e09.q1c-rev |
|  |  |  | tryp_IXb-334g12.p1c-rev |
|  |  |  | tryp_XI-1117h04.q1k |
|  |  |  | tryp_IXb-274b10.q1c-rev |
|  |  |  | tryp_IXb-1e12.q1c-rev |
|  |  |  | tryp_IXb-282d11.q1c |
|  |  |  | tryp_IXb-342g05.p1c-rev |
|  |  |  | tryp_IXb-375c10.q1c-rev |
|  |  |  | tryp_XI-1117h04.p1k-rev |
|  |  |  | tryp_IXb-125h06.q1c-rev |
|  |  |  | tryp_IXb-282d11.p1c-rev |
| tryp_XI-325h02_assembly | 77 | 3' incomplete | tryp_XI-1024b10.p1k-rev |
|  |  |  | tryp_XI-1128e06.p1kw-rev |
|  |  |  | tryp_XI-1139e06.q1k |
|  |  |  | tryp_XI-238f01.p1c-rev |
|  |  |  | tryp_XI-325h02.q1k-rev |
|  |  |  | tryp_XI-325h02.q1ka-rev |
|  |  |  | tryp_XI-330f02.q1k |
|  |  |  | tryp_XI-330f02.q1ka |
|  |  |  | tryp_XI-912a01.p1k-rev |
| 927mc_III-7e05_assembly | 79 | 3' incomplete, not contiguous | 927mc_III-7e05.p1k |
|  |  |  | 927mc_III-7e05.q1k |
| 927mc_VI-4a12_assembly | 80 | 3' incomplete | 927mc_VI-4a12.p1k |
|  |  |  | 927mc_VI-5d03.p1k |
|  |  |  | 927mc_V-8d11.p1k |
|  |  |  | 927mc_V-1d03.q1k-rev |
|  |  |  | 927mc_VI-12c01.q1k-rev |
|  |  |  | 927mc_VI-9c10.q1k |
|  |  |  | 927mc_VI-11g04.p1k-rev |
| 927mc_VI-4g03_assembly | 81 |  | 927mc_VI-4g03.q1k |
|  |  |  | 927mc_V-6d03.q1k |
|  |  |  | 927mc_VI-2g12.q1k |
|  |  |  | 927mc_V-6d05.p1k |
|  |  |  | 927mc_VI-10e07.q1k |
| 927mc_VI-6c05_assembly | 82 |  | 927mc_VI-6c05.p1k-rev |
|  |  |  | 927mc_VI-6c05.q1k-rev |
|  |  |  | 927mc_VI-4a05.p1k-rev |
|  |  |  | 927mc_VI-5e11.p1k |
|  |  |  | 927mc_VI-5e11.q1k-rev |
|  |  |  | 927mc_VI-5h02.p1k |
|  |  |  | 927mc_VI-7e06.q1k-rev |
|  |  |  | 927mc_VI-13c03.q1k |
| 927mc_VII-13h07_assembly | 83 | 3' incomplete | 927mc_VII-13h07.p1k-rev |
|  |  |  | 927mc_VII-13h07.q1k-rev |
|  |  |  | 927mc_VII-5h01.p1k-rev |
|  |  |  | 927mc_VII-7g03.q1k-rev |
|  |  |  | 927mc_VIII-1f08.q1k-rev |
|  |  |  | 927mc_VII-11c05.p1k |
|  |  |  | 927mc_VI-11f11.p1k-rev |
| 927mc_VIII-14d09_assembly | 84 |  | 927mc_VIII-14d09.p1k |
|  |  |  | 927mc_VIII-14d09.q1k |
|  |  |  | 927mc_IV-5h11.p1k |
|  |  |  | 927mc_VII-5a05.q1k-rev |
| tryp_XI-224e03_assembly | 85 | not contiguous | tryp_XI-224e03.q1c |
|  |  |  | tryp_XI-224e03.p1c-rev |
|  |  |  | tryp_XI-943h03.q1k-rev |
| tryp_IXa-28d11_assembly | 86 |  | tryp_IXa-28d11.q1c-rev, |
|  |  |  | tryp_IXa-17g02.p1c-rev |
|  |  |  | tryp_X-316d12.q1c-rev |
| tryp_IXb-338f09_assembly | 87 |  | tryp_IXb-68a11.q1c-rev |
|  |  |  | tryp_IXb-18c04.q1c-rev |
|  |  |  | tryp_IXb-257c04.q1c |
|  |  |  | tryp_IXb-257g03.q1c-rev |
|  |  |  | tryp_IXb-296f08.p1c |
|  |  |  | tryp_IXb-338f09.p1c |
|  |  |  | tryp_IXb-302g08.p1c |
| tryp_X-187a04_assembly | 87 | 5' incomplete | tryp_X-187a04.p1c |
|  |  |  | tryp_X-187a04.q1c-rev |
|  |  |  | tryp_X-402h07.p1c |
| tryp_X-274d02_assembly | 88 | 3' incomplete, not contiguous | tryp_X-350d12.q1c-rev |
|  |  |  | tryp_X-246a07.p1c-rev |
|  |  |  | tryp_X-440d01.q1c |
|  |  |  | tryp_X-274d02.p1c-rev |
|  |  |  | tryp_X-135c02.q1c-rev |
| tryp_XI-1087c04.q1k-rev | 89 | 5' and 3' incomplete | tryp_XI-1087c04.q1k-rev |
| tryp_IXa-3a03_assembly | 91 | 5' incomplete, not contiguous | trypB62c6.q1t-rev |
|  |  |  | tryp_IXa-3a03.p1c |
|  |  |  | tryp_IXa-3a03.q1c-rev |
|  |  |  | tryp_IXb-89d01.p1c-rev |
|  |  |  | tryp_IXb-89d01.q1c |
|  |  |  | tryp_XI-982g12.q1k |
| tryp_XI-1077d06_assembly | 93 | 3' incomplete | tryp_XI-1077d06.q1k |
|  |  |  | tryp_XI-170f03.p1k |
| tryp_IXb-321a12_assembly | 94 | 3' incomplete | tryp_IXb-321e12.q1c |
|  |  |  | tryp_IXb-321e12.p1c-rev |

^a^The ‘notes’ column provides GenBank accession numbers for those donors cloned and sequenced from gDNA.

^b^When assembling reads, mismatches between the reads were treated as follows. If the mismatch occurred towards the ends of a read, it was considered to be the result of poor quality sequencing, and was corrected in favour of the other read(s). If the mismatch occurred in the middle of the read, but more than one other read did not contain the mismatch, it was corrected in favour of the other reads. If the mismatch occurred in the middle of the read, and there was only one other read with which to compare it, the mismatch was corrected in favour of the corresponding clone sequence. Read sequences were obtained from the following files obtained from <ftp://ftp.sanger.ac.uk/pub/databases/T.brucei_sequences/> : T_brucei_chrIXa_reads.20Oct2001, T_brucei_chrIXa_reads.29May03, T_brucei_chrIXb_reads.20Oct2001, T_brucei_chrIXb_reads.29May03, T_brucei_chrI_reads.03Feb2000, T_brucei_chrXI_reads.V1_17Oct2002, T_brucei_chrXI_reads.V2_14Mar2003.fas, T_brucei_chrXI_reads.V3_11Jul03.fas, T_brucei_chrX_reads.03Mar2003, T_brucei_chrX_reads.20Oct2001, T_brucei_chrX_reads.30Apr2001, T_brucei_reads.21Dec1999, TbchrIXa_reads_26May04.fas, TbchrIXb_reads_26May04.fas, Tb927_IC_II_reads.fas, Tb927_IC_I_reads.fas, 177bp_repeat.dnaTb927_MC_cons.dna.crunch, 177bp_repeat.dnaTb927_MC_test_reads.dna.crunch, 927MC_readsII.fas, 927MC_readsIII.fas, 927MC_readsIV.fas, 927MC_readsV.fas, 927MC_readsVI.fas, 927MC_readsVII.fas, 927mcIII_v1.cons, 927mcII_v1.cons, 927mcIV_v1.cons, 927mcIX_v1.cons, 927mcVIII_v1.cons, 927mcVII_v1.cons, 927mcVI_v1.cons, 927mcV_v1.cons, Tb927MC_telo1_contigs.fas, Tb927MC_telo2_contigs.fas, Tb927MC_telo2_v2contigs.fas, Tb927MC_telo9_contigs.fas, Tb927MC_telo9_v2contigs.fas, Tb927MCtelo_1_test.fas, Tb927MCtelo_2_test.fas, Tb927MCtelo_9_test.fas, Tb927_MC.cons, Tb927_MCIX_contigs_6Mar08.fas, Tb927_MC_III_reads.fas, Tb927_MC_II_reads.fas, Tb927_MC_IV_reads.fas, Tb927_MC_IX_reads.fas, Tb927_MC_VIII_reads.fas, Tb927_MC_VII_reads.fas, Tb927_MC_VI_reads.fas, Tb927_MC_V_reads.fas, Tb927_MC_test_reads.fas, Tb927_MC_testreads_newlib.fas, uniprot-1-xTb927_MC_cons.dna.crunch, uniprot-1-xTb927_MC_test_reads.dna.crunch, TPunknown_May06.cons, Tbrucei_extrachr_testreads_Dec05.fas, chr_unknown_test_reads_Jan06.fas
